# Myocarditis complicated by massive right ventricular thrombus and extensive pulmonary embolism: A case report

**DOI:** 10.3389/fsurg.2022.924366

**Published:** 2022-08-16

**Authors:** Xiao-juan Jiang, Wei-yi Zhang

**Affiliations:** Department of Anesthesiology, West China Hospital, Sichuan University, Chengdu, China

**Keywords:** myocarditis, right ventricular thrombus, pulmonary embolism, case report, surgery

## Abstract

An intracardiac thrombus may develop as a consequence of myocarditis, and in rare cases, a dominantly right ventricular thrombus develops, which may impair cardiac function and even cause life-threatening cardiovascular events. We report a 24-year-old man presented with recurrent episodes of palpitation and precordial discomfort after catching a cold 2 months ago. Transthoracic echocardiography (TTE) and computed tomography pulmonary angiogram (CTPA) revealed a mass attached to the apex of the right ventricle and extensive bilateral pulmonary artery emboli. There was no indication where the thrombi originated from in this young patient without any underlying disease except myocarditis. Pulmonary endarterectomy and embolectomy of pulmonary arteries and right ventricle were performed. Postoperative pathological results confirmed the presence of fibrinous necrosis and hemosiderin deposition. The formation of an intraventricular thrombus is closely related to myocarditis, which can affect individuals of all ages, but especially young people. Thus, patients with myocarditis should be closely monitored and followed up because of the increased risk of extensive thrombosis.

## Introduction

Myocarditis is an inflammatory disease of cardiac tissue that may be caused by infections, systemic disease, exposure to drugs or toxic substances, or abnormal immunoreactivity (1). Its clinical presentations are heterogeneous and evolving, varying from asymptomatic, through mild symptoms such as atypical chest pain and palpitations, to life-threatening cardiovascular events, such as heart failure, refractory arrhythmias and even cardiogenic shock (2, 3). An intracardiac thrombus may develop as a consequence of myocarditis and is an important prognostic factor. A thrombus only in the right ventricle is uncommon, especially when not associated with deep venous thrombosis of the lower extremities. Here, we present an unusual case of a right ventricular thrombus complicated by widespread bilateral pulmonary embolism occurring after myocarditis in a young patient without any underlying disease.

## Case report

**Figure 1:**
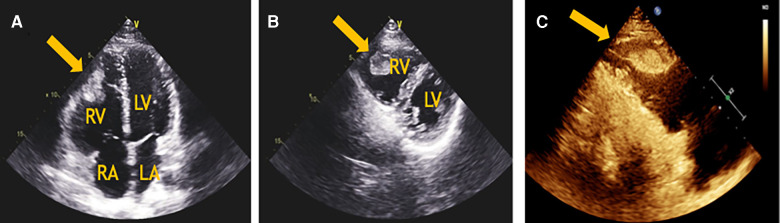
(**A**) The apical 4-chamber view of transthoracic echocardiography revealed a mass in the RV and a moderately dilated right heart. (**B**) The parasternal short-axis view of transthoracic echocardiography revealed a solid, poorly mobile mass attached to the apex of the RV. (**C**) The myocardial perfusion examination suggested moderate to low perfusion. LA, left atrium; LV, left ventricle; RA, right atrium; RV, right ventricle.

**Figure 2:**
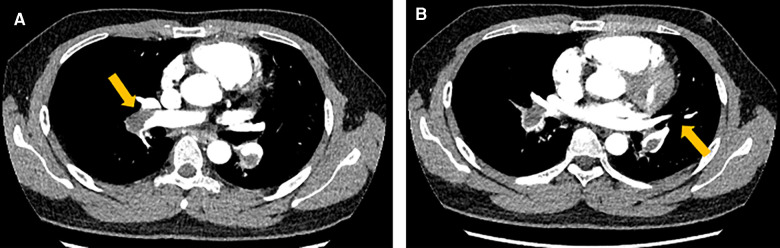
The computed tomography pulmonary angiogram (CTPA) presented extensive emboli and signs of bilateral pulmonary artery stenosis and filling defects, which were indicated by the arrows both in image **A** and **B**.

A 24-year-old Chinese man who was a graduate student presented to the Department of Cardiovascular Surgery complaining of recurrent episodes of palpitation and precordial discomfort, which had been worsened by exertion two months ago after catching a cold. The initial symptom was an occasional cough, which was not taken seriously and he didn't take any medication at the beginning. Gradually, the patient experienced occasional chest tightness and chest pain. No syncope, dizziness, dyspnoea, or lower limb oedema were observed. No oral ulcers, genital ulcers or ophthalmitis were detected. The nucleic acid test for COVID-19 was negative. The initial clinical consideration was myocarditis, and he was treated with oral Betaloc 47.5 mg qd. and trimetazidine 20 mg tid. for about two weeks. The patient's personal and family history was largely disease-free. Physical examination revealed a resting tachycardia of 130 beats per minute (bpm) and a stable haemodynamic with blood pressure 123/91 mm Hg. His temperature was 36.5°C and respiratory rate (RR) was 20 breaths per min with oxygen saturation of 96% on room air. Cardiovascular examination was remarkable for the heart border expanded to the left bottom. The heart rhythm was steady, and no murmur was heard in each valve area. An electrocardiogram revealed sinus tachycardia and T-wave changes. The systolic function of the left ventricle (LV) was normal (EF 66%), and the systolic function of the right ventricle (RV) was at the lower threshold (TAPSE 14 mm). No additional treatment was used to improve the function of RV. Transthoracic echocardiography (TTE) revealed a moderately dilated right heart (RA 52 mm, RV 31 mm) and mild tricuspid regurgitation. A mass having a solid, wide base and poor mobility measuring 39 mm × 21 mm was attached to the apex of the RV, causing moderate to severe RV hypokinesis (Figures 1A,B*)*. Myocardial perfusion examination suggested moderate to low perfusion (Figure 1C). Subsequently, CTPA revealed extensive bilateral pulmonary emboli in the right pulmonary artery, left lower pulmonary artery and multiple branching arteries (Figure 2). Bilateral lower extremity venous Doppler ultrasound did not identify any evidence of embolus. No outliers were observed in the preoperative laboratory tests as presented: RBC 5.62 × 10^12^/L, PLT 298 × 10^9^/L, and WBC 5.10 × 10^9^/L. The results of the myocardial markers were also within the normal range with CK-MB at 0.78 ng/ml (<4.94 ng/ml), myoglobin <21 ng/ml, troponin T 6.6 ng/L (0–14 ng/L), and urinary sodium 76 ng/L (0–88 ng/L). CRP was 6.37 mg/L, which was a bit higher than the reference range (<5 mg/L). In addition, prothrombin, renal function and lipid levels were all within the normal range. The anticardiolipin antibody tests all showed negative: IgA (−), IgG (−) and IgM (−). The test for lupus antibody, represented as LA1/LA2, yielded 1.31 (0.8–1.2). Screening for thrombosis and gene sequencing demonstrated that protein S activity increased by 133.7% (60–130%), factor VIII activity increased by 165.9% (60–150%), and multi-point dilution VIII (1:1), (1:2) and (1:4) increased by 164.1%, 166.8% and 165.1%, respectively. The activity of protein C, antithrombin III, factor IX and multi-point dilution IX (1:1), (1:2) and (1:4) were all normal. PET/CT indicated that there was no increase in glucose metabolism in the RV mass, which means that it was considered as a benign tumour.

**Figure 3:**
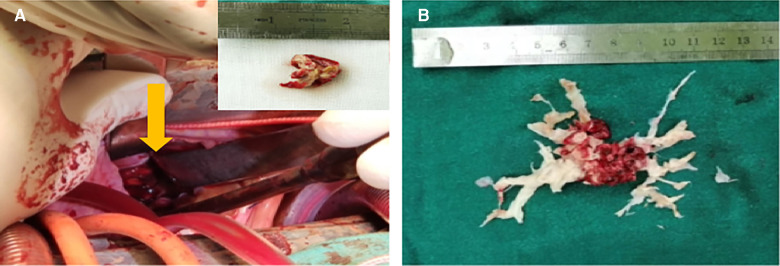
(**A**) The right ventricular mass found during the operation. (**B**) The denuded pulmonary artery intima during surgery.

Due to the extensive pulmonary emboli and the lack of any evidence of malignancy, surgical treatment is recommended after multidisciplinary consultations. Pulmonary endarterectomy and embolectomy of pulmonary arteries and the right ventricle were performed under general anaesthesia and cardiopulmonary bypass. A mass measuring 1.8 × 1.0 mm was found in the RV. The bilateral pulmonary trunk and some branches were also involved. Fortunately, the pulmonary and tricuspid valves were intact. The exfoliated pulmonary intima and the RV thrombus are shown in Figure 3. The pathology results on specimens from the RV and pulmonary arteries showed fibrinous necrosis and hemosiderin deposition, indicating a thrombotic component.

The operation was successful and the patient was discharged on the 10th day after surgery. He was treated with permanent anticoagulant therapy by taking warfarin orally 2.5 mg qd. and the international normalized ratio (INR) was monitored intermittently. Follow-up was performed at three and six months, and repeat TTE showed complete resolution of the thrombus. The patient reported no discomfort and exercise tolerance was restored.

## Discussion

Right ventricular thrombi (RVT) are uncommon, which are seen in less than 4% of patients with pulmonary embolism (PE) according to the 2019 ESC Guidelines for Acute Pulmonary Embolism, but the incidence of pulmonary embolization from RVT has not been clearly documented (4). This case was remarkable for the unusual location of the thrombus in the right ventricle and the complication of widespread bilateral PE, which made the diagnostic process particularly difficult and complex.

A provisional diagnosis of myocarditis with RV thrombus was made. There was no common risk factor of thromboembolism such as deep vein thrombosis or a long bed-ridden period. The moderator band, papillary muscle, and coarse trabeculations in RV make the diagnosis of right ventricular thrombus challenging (5). A thrombus in the right heart usually originates from deep vein thrombosis, and other common causes include atrial fibrillation and intracardiac foreign bodies, such as prosthetic valves, pacemakers, and catheters (6, 7). Besides, some rare causes of RVT formation have been reported in hypercoagulable states (factor V deficiency, protein C and protein S deficiencies), Bechet's disease, antiphospholipid syndrome (APLAS), eosinophilia, primary thrombocytopenia, RV infarction, and various cardiomyopathies such as dilated or alcoholic cardiomyopathy and arrhythmogenic right ventricular dysplasia (8–10), but RVT was less frequently described in myocarditis.

In this patient, the anticardiolipin antibody tests were negative, while the test for lupus antibody was initially positive, suggesting a possible diagnosis of antiphospholipid antibody syndrome, but this was not confirmed in the further test. Given the absence of hypercoagulability and a possible history of associated disease, the only cause traceable to this patient was myocarditis. However, the embolus was not found in the patient's cardiac colour ultrasound examination at the first visit, but was found in the examination two months later, suggesting that the frequency of cardiac ultrasound examination should be increased for patients with myocarditis, especially when symptoms are aggravated.

Several conditions may contribute to the high risk of thrombus formation in myocarditis (9–13). The first is acquired or inherited thrombophilia (14, 15). As is known, a mutation in the methylene tetrahydrofolate reductase gene (C677T, A1298C) and hyperhomocysteinemia are important risk factors for thrombosis, which may cause damage to the endothelium and affect thrombocytes and coagulation factors (16). Secondly, viral myocarditis can promote myocardial tissue factor (TF) expression and procoagulant activity, which leads to hyperactivation of the coagulation system (17). Some evidence suggests that virus-triggered immune-mediated reactions are the principal cause of cardiomyocyte injury. A series of hospitalized coronavirus disease (COVID-19) patients revealed that the rate of acute cardiac injury ranged from 7% to 27% with poor diagnosis (18). They had significantly higher levels of D-dimer and fibrin degradation product (FDP) (19). Also, because myocarditis is an inflammatory disease of the cardiac muscle, the inflammatory cytokines produced may activate the coagulation cascade resulting in thrombosis (20). Thus, the significance of myocarditis should be taken seriously in light of the clinical scenario. Myocarditis can even be associated with the formation of multi-compartmental thrombi (21, 22). As Thuny et al. reported using 2-dimensional and real-time 3-dimensional echocardiography, massive biventricular thrombi were found as a consequence of myocarditis (23). Atas et al. presented two cases of multiple intracardiac thrombus formation attributable to inflammation and hypercoagulable states (24).

Right ventricular embolism can easily develop into PE, and there are no evidenced-based guidelines for the treatment of PE complicated by a right ventricular thrombus (25). It was reported that patients with PE and right heart thrombus experienced increased mortality, ranging from 16.7% to 50%, which could be as high as 80–100% without treatment (26, 27). A multidisciplinary consultation was necessary in this case. The preferred treatment is surgical resection of the embolic intima and part of the tunica media that have undergone fibrous reorganization in the pulmonary artery. Long-term anticoagulant therapy and regular follow-up are required after surgery. Thus, we need to pay greater attention to right heart embolism and take early and timely intervention measures. Transthoracic echocardiography is a simple and intuitive examination for early detection.

## Conclusions

In conclusion, the formation of an intraventricular thrombus is closely related to myocarditis, which can affect individuals of all ages, but especially young people. The optimal treatment for those patients with right heart thrombi remains uncertain. Despite the successful outcome of this case, patients with myocarditis should be followed up carefully and additional studies on whether anticoagulant therapy should be applied earlier are needed. A comprehensive management system with guidelines should be established for the prevention and treatment of intraventricular thrombus caused by myocarditis.

## Data Availability

The raw data supporting the conclusions of this article will be made available by the authors, without undue reservation.
